# Diagnostic Challenges of Dermatofibrosarcoma Protuberans (DFSP), a Rare Spindle Cell Tumor of Breast

**DOI:** 10.7759/cureus.20643

**Published:** 2021-12-23

**Authors:** Sanobar Yasmeen Mohammed, Qandeel Sadiq, David Mcgregor, Farhan Khan

**Affiliations:** 1 Department of Pathology and Laboratory Medicine, Orlando Regional Medical Center, Orlando, USA; 2 Department of Pathology and Laboratory Medicine, The University of Tennessee Health Science Center, Memphis, USA

**Keywords:** locally aggressive, recurrent, spindle cell lesion, breast, dermatofibrosarcoma

## Abstract

Dermatofibrosarcoma protuberans (DFSP) is a low-grade, fibroblastic tumor that is rarely seen in the breast with only a few cases reported in the literature. DFSP poses a diagnostic challenge as there is significant cytomorphological overlap with other spindle cell lesions. We report a case of a 42-year-old female who presented with a nodule in the right breast. Histology revealed a hypercellular lesion composed of spindle cells infiltrating the fat. A diagnosis of spindle cell lipoma was made. However, two years later, the patient developed a recurrent mass in the right breast that was histologically consistent with DFSP with a predominant myxoid stroma obscuring the characteristic storiform architecture with a focal component of giant cell fibroblastoma. A careful histomorphological examination is warranted as DFSP tends to recur if not completely excised.

## Introduction

Dermatofibrosarcoma protuberans (DFSP) is a rare soft tissue tumor that is locally aggressive and has a high recurrence rate on excision. It is also characterized by slow, infiltrative growth and is misdiagnosed frequently. DFSP is diagnosed mostly in people aged between 30 to 50 years, with slight male preponderance [1]. It mostly occurs in the trunk and extremities. It occurs rarely in the breast and diagnosis can be challenging [2]. This article highlights a rare case of recurrent DFSP in the breast that was treated with repeated excision. 

This article was previously presented as an abstract at the College of American Pathologists 2020 Annual Meeting (CAP20 Virtual) and published (as such) as part of the Abstracts and Case Studies for the College of American Pathologists 2020 Annual Meeting (CAP20 Virtual) in the Archives of Pathology & Laboratory Medicine on September 09, 2020 [3]. 

## Case presentation

**Figure 1:**
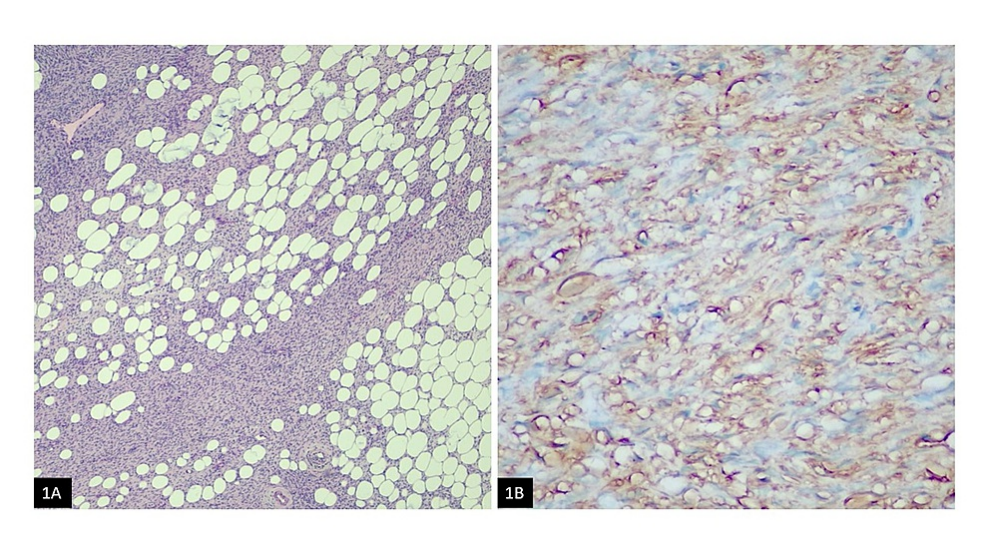
(A) Bland spindle cells infiltrating the fibroadipose tissue (H&E); (B) Positive CD34 staining H&E: Haemotoxylin and Eosin

**Figure 2:**
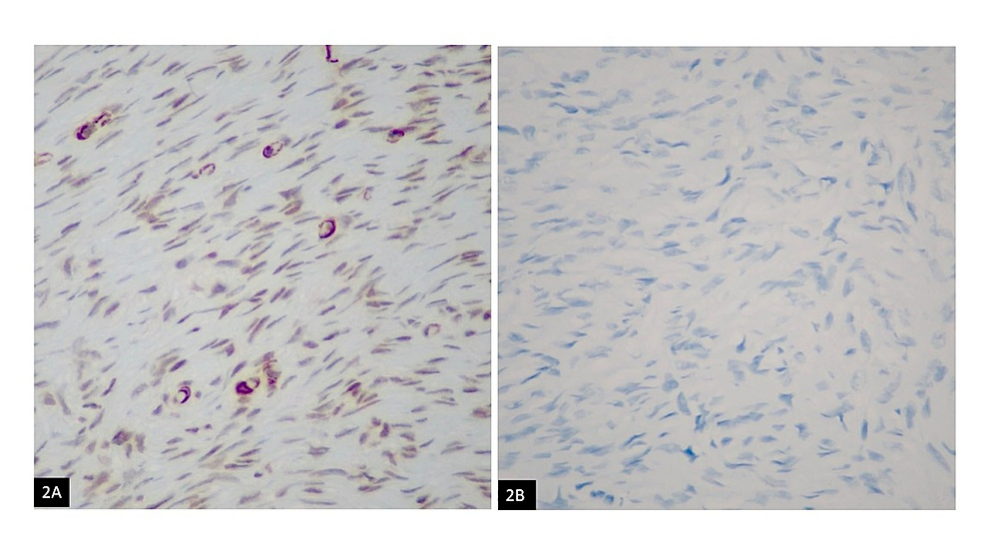
(A) Positive smooth muscle actin (SMA); (B) Negative cytokeratin (CK) staining

**Figure 3:**
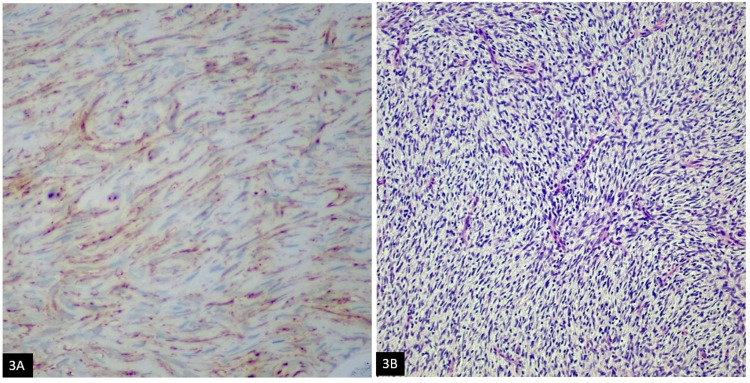
(A) Cytoplasmic beta-catenin staining; (B) Uniform spindle cells arranged in a storiform pattern (H&E) H&E: Haemotoxylin and Eosin

A 42-year-old female presented with a palpable right breast lesion. There was no history of any associated pain, nipple discharge, or lumps in the axilla. On mammography, the breasts were composed of scattered fibro-glandular densities. Adipose tissue replacing normal parenchyma was seen in the area of a palpable breast lesion. No discrete mass, area of spiculation, or malignant microcalcification was identified. Targeted right breast ultrasound revealed a hyperechoic nodule measuring 0.8 x 1.0 cm in size at 6 o’clock, 9 cm from the nipple in the right breast. Over the option of a six-month interval follow-up versus biopsy, the patient elected to proceed with the biopsy, which demonstrated hypercellular fibrous tissue infiltrating adipose tissue. Excision was recommended for complete histopathological evaluation. The patient underwent a right breast excisional biopsy. Grossly, a 0.3 x 0.2 cm tan-white indurated mass was seen abutting the superior margin. Histologically, there was a bland spindle cell lesion, identical to the previous biopsy. The uniform spindle cells infiltrating the surrounding stroma and fibro-adipose tissue (Figure 1A) were identified. No areas of cytological atypia, necrosis, or mitosis were seen. The tumor was present at the inked superior excision margin. Immunohistochemical analysis showed the spindle cells were positive for CD34 (Figure 1B), and smooth muscle actin (SMA) (Figure 2A) immunostains. Pan keratin (Figure 2B), Cytokeratin-7, Cytokeratin 34BE12, Cytokeratin-8/18, p63, and Desmin were all negative. Beta-catenin (Figure 3A) showed a cytoplasmic staining pattern. Based on the morphologic and immunophenotypic expression, a diagnosis of spindle cell lipoma was rendered.

**Figure 4:**
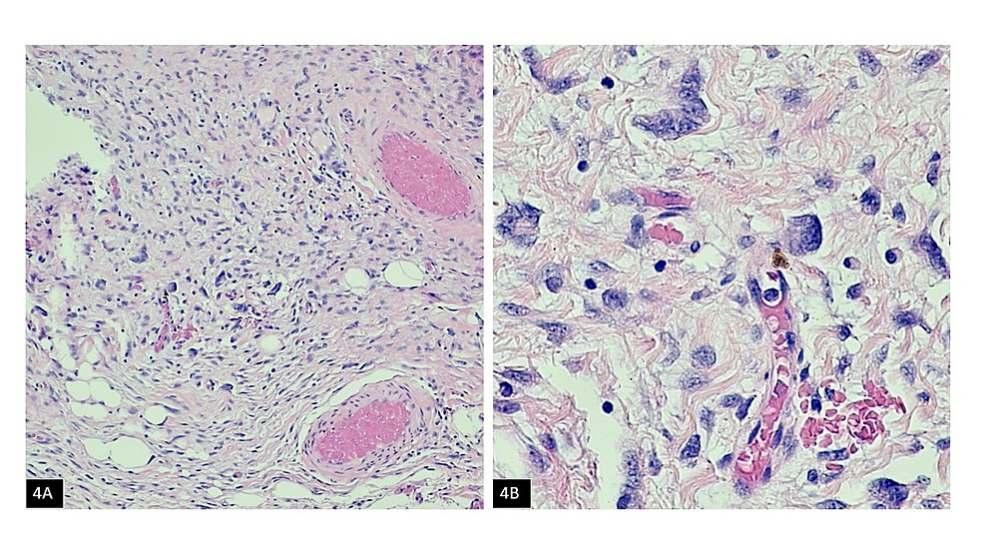
Giant cell fibroblastoma component (H&E): (A) Low power, (B) High power H&E: Haemotoxylin and Eosin

Follow-up mammography and ultrasonography at four-month intervals demonstrated a residual lobulated hyperechoic mass measuring at least 1.2 cm at the scar site. Due to the persistent mammographic abnormality, the patient was scheduled for surgical re-excision. No distinct mass was identified on gross examination. Microscopic evaluation revealed biopsy site changes. No atypia or malignancy was identified. Two years following the re-excision, the patient presented with a new palpable right breast mobile mass, 1.0 x 1.0 cm at 6 o'clock, 9 cm from the nipple. Imaging showed a recurrent tumor at the site of the incision. On mammography and targeted ultrasound, an oval mass with high density circumscribed borders was found at 6 o'clock in the skin surface close to the posterior third of the chest wall, measuring 1.6 cm in diameter. Right breast lumpectomy was performed. Gross examination revealed a 1.6 x 1.6 x 1.2 cm tan-pink, ill-defined, subdermal mass. Histology showed an infiltrative lesion in the dermis composed of uniform spindled cells. Spindle cells were arranged in a vague storiform pattern (Figure 3B) in a background of the myxoid stroma. Spindle cells showed infiltration of subcutaneous fat with a characteristic honeycomb/swiss cheese appearance. Nuclei were elongated and wavy. A focal component of giant cell fibroblastoma was also present (Figures 4A-4B). There was minimal cytological atypia and mitotic figures were sparse (<5 per 10 HPF). The overlying epidermis was unremarkable. Margins of resection were free of DFSP. The previous lesion was also carefully reviewed again and a subtle DFSP was identified.

## Discussion

Primary sarcomas of the breast are very rare mesenchymal tumors that account for about 0.2% to 1.0% of all breast cancers [2]. DFSP is seen mostly in patients in the age group of 30-40 years, with a slight male preponderance [4]. The most common sites for the development of DFSP are the trunk (42% to 72%) and extremities (16% to 30%) but the breast is a very rare site. It has a high rate of local recurrence, but in 5% of the cases, it undergoes a fibrosarcomatous transformation [2]. The recurrence rate is accounted for due to tentacle-like projections of the tumor through fat, fascia, and muscle. Distant metastases such as pulmonary and tumor-related deaths are very rarely related to DFSP. The five-year survival rate of patients with local DFSP is up to 99% [5-9].

According to genetic studies, more than 90% of DFSP has specific chromosomal translocation (chromosomes 17 and 22) which results in constitutive production of platelet-derived growth factor receptor alpha (PDGFR-α) and overt growth of DFSP. A novel *COL6A3*-*PDGFD* fusion variant in dermatofibrosarcoma protuberans appears to have a predilection for the breast. The chimeric proteins arising from *COL6A3-PDGFD* and *COL1A1*-*PDGFB* fusion genes both retain the PDGF domain which in turn activates the platelet-derived growth factor receptor beta (PDGFR-β), which is responsible for recurrent growth [6,7].

Ultrasound and mammography in this patient defined an oval mass with high density circumscribed borders developed two years after detecting architectural changes in the same site after surgery. Histologically, the lesion in the patient was consistent with DFSP involving the dermis and subcutaneous soft tissue, with a predominant myxoid stroma obscuring the characteristic storiform architecture. Focally, the lesion showed patternless spindled cells admixed with multinucleated giant cells representing a component of giant cell fibroblastoma. Tumor cells were reactive to CD34, and beta-catenin showed cytoplasmic staining. Tumor cells were non-reactive to CK Oscar, Melan-A, actin, desmin, S100, CD31, and MDM2.

According to Oscan et al. in the case they reported, DFSP also shows CD34 positivity and SMA positivity, which distinguishes it from leiomyoma and dermatofibroma. Sometimes histological variants amount to some confusion in diagnosis. The histological variants can be pigmented; with focal myofibroblastic areas; myxoid, with sclerosing nodules; atrophic, or granular cell, fibrosarcoma-like changes; or giant cell fibro blastoma-like areas [8-12]. Differential diagnosis includes fibroadenomas and phyllodes tumors as the most common, followed by pseudo angiomatous stromal hyperplasia, hamartoma, adenomyoepithelioma, metaplastic carcinoma, and fibromatosis [7,9,10].

Surgery is the preferred treatment for DFSP. As it has a high recurrence rate, treatment is complete surgical excision with wide margins (>3 cm) or Mohs micrographic surgery [5,13]. Wide excision may not be preferred in the breast for the fear of closure difficulties [7]. A multi-disciplinary approach should be done to close the surgical excision [11]. The patient underwent surgery three times for the same lesion, which defines the nature of the lesion. Long-term follow-up is required with surveillance mammography [7].

## Conclusions

In conclusion, DFSP of the breast is uncommon. It usually mimics a benign primary breast mass both clinically and radiographically. The spectrum of differential diagnosis ranges from benign to malignant spindle cell lesions. Characteristic histological and immunohistochemical features will aid us in the diagnosis of DFSP. Biopsy from non-representative tissue may mislead us sometimes. Surgical excision is the highly recommended mode of treatment to prevent its recurrence. Patients require follow-up with surveillance mammography as distant metastasis, though rare, has been reported. 
